# WRKY2/34–VQ20 Modules in *Arabidopsis thaliana* Negatively Regulate Expression of a Trio of Related MYB Transcription Factors During Pollen Development

**DOI:** 10.3389/fpls.2018.00331

**Published:** 2018-03-19

**Authors:** Rihua Lei, Zhenbing Ma, Diqiu Yu

**Affiliations:** ^1^Key Laboratory of Tropical Plant Resources and Sustainable Use, Xishuangbanna Tropical Botanical Garden, Chinese Academy of Sciences, Kunming, China; ^2^College of Life Sciences, University of Chinese Academy of Sciences, Beijing, China

**Keywords:** *WRKY2*, *WRKY34*, *MYB97*, *MYB120*, male gametogenesis, *Arabidopsis*

## Abstract

Male gametogenesis in plants is tightly controlled and involves the complex and precise regulation of transcriptional reprogramming. Interactions between WRKY proteins and VQ motif-containing proteins are required to control these complicated transcriptional networks. However, our understanding of the mechanisms by which these complexes affect downstream gene expression is quite limited. In this study, we found that *WRKY2* and *WKRY34* repress *MYB97*, *MYB101*, and *MYB120* expression during male gametogenesis. *MYB* expression was up-regulated in the *wrky2-1 wrky34-1 vq20-1* triple mutant during male gametogenesis. The expression levels of six potential targets of the three *MYBs* increased the most in the *wrky2-1 wrky34-1 vq20-1* triple mutant, followed by the *wrky2-1 wrky34-1* double mutant, compared with in wild-type. Yeast one-hybrid and dual luciferase reporter assays indicated that WRKY2 and WRKY34 recognized the *MYB97* promoter by binding to its W-boxes. *MYB97* overexpression caused defects in pollen germination and pollen tube length, which impacted male fertility. Thus, WRKY2/34–VQ20 complexes appear to negatively regulate the expression of certain *MYBs* during plant male gametogenesis.

## Introduction

In flowering plants, the male gametophyte (pollen) delivers the male gametes (sperm nuclei) to the embryo sac for double fertilization (McCormick, 2004; Berger and Twell, 2011). The male gametophytic life cycle can be divided into two consecutive phases, developmental and progamic (Borg and Twell, 2011). The developmental phase consists of microsporogenesis and microgametogenesis (Sanders et al., 1999). During this phase, the uninucleate microspore undergoes pollen mitosis I to create bicellular pollen containing a large vegetative cell and a generative cell. Then the generative cell generates two sperms after pollen mitosis II (tricellular pollen), which ends with pollen maturation (mature pollen) (McCormick, 2004). The progamic phase initiates after the pollen grain lands on the stigma, and includes pollen germination, pollen tube growth through the transmitting tissue, sperm nuclei discharge into the embryo sac, and finally the fusion of the male and female gametes (Borg et al., 2009; Hafidh et al., 2016). The two phases are highly transcriptionally regulated (Honys and Twell, 2004; Rutley and Twell, 2015); however, little is known regarding the transcription factors involved in the temporal and spatial regulation of pollen development (Ma and Sundaresan, 2010; Berger and Twell, 2011; Hafidh et al., 2016).

The precise and dynamic regulation of male gametogenesis requires the involvement of multiple transcription factors, and the expression of 607 transcription factors was reported in *Arabidopsis thaliana* during pollen development (Honys and Twell, 2004). For example, three pollen-expressed *MYB* genes, *MYB97*, *MYB101* and *MYB120*, encode highly similar amino acid sequences and play crucial roles in controlling the growth of the pollen tube and its interactions with the synergids. *MYB97*, *MYB101*, and *MYB120* localize in pollen tube nuclei and induce at pollen growth through pistil tissue. The loss of function of these genes does not lead to defects in pollen development, pollen germination, or pollen tube growth, but the pollen tubes fail to burst and release the pollen sperm cells after entering the pistil tissue. Further analyses revealed that the pollen tubes of the *myb97 myb101 myb120* triple mutant exhibit uncontrolled growth and fail to discharge the pollen sperm cells because of the altered expression of a suite of pollen tube-expressed genes (Leydon et al., 2013; Liang et al., 2013). However, the mechanisms regulating the expression levels of *MYB97*, *MYB101*, and *MYB120* during male gametogenesis are still poorly understood.

The WRKY protein superfamily is among the 10 largest families of transcription factors in higher plants, and it controls a plethora of processes, including biotic and abiotic stress responses, and plant development (Chen et al., 2009; Pandey and Somssich, 2009; Rushton et al., 2010; Castrillo et al., 2013). In *Arabidopsis*, *WRKY34* is specifically expressed in the male gametophytes and involved in the early stages of their development (Honys et al., 2006; Guan et al., 2014). We previously have demonstrated that *WRKY34* expression is induced significantly by cold treatment in the wild-type. Mature pollen grains of *WRKY34* loss-of-function mutant, *wrky34-1* (termed *w34-1*), are more viable than those of wild-type under cold treatment conditions, having increased germination efficiency levels and pollen tube growth rates in plant (Zou et al., 2010). Jiang and Yu (2009) found that the WRKY2 transcription factor plays a critical role in controlling seed germination and post-germination development in an abscisic acid-dependent manner (Jiang and Yu, 2009). Moreover, Guan et al. (2014) reported that WRKY34 is temporarily phosphorylated by MPK3 and MPK6 kinases at the early stages of male gametogenesis. *WRKY2*, which is closely related to *WRKY34*, is also expressed in pollen, and it functions redundantly with *WRKY34* in pollen development, pollen germination, and pollen tube growth (Guan et al., 2014). A pollen-specific expressed VQ motif-containing protein, VQ20, interacts with WRKY34 and WRKY2 to modulate the function of WRKYs in pollen development. WRKY and VQ20 loss-of-function enhances the defects in pollen development, pollen germination, and pollen tube growth of *wrky34 wrky2* double mutant. A microarray analysis indicated that *WRKY2*, *WRKY34*, and *VQ20* co-modulate multiple genes involved in pollen development, pollen germination, and pollen tube growth (Lei et al., 2017). Nevertheless, the downstream target genes and mechanisms underlying the *WRKY2/34–VQ20* mediated processes are still not fully understood.

In this study, *WRKY2* and *WKRY34* repressed the expression of *MYB97*, *MYB101*, and *MYB120* during male gametogenesis. *MYB* expression was up-regulated in the *w2-1 w34-1 vq20-1* triple mutant relative to the wild-type during male gametogenesis. WRKY2 and WRKY34 bind to W-boxes in the promoter of *MYB97*, as revealed by yeast one-hybrid and dual luciferase reporter assays, and overexpression of *MYB97* leads to male fertility defects. This study indicated that WRKY2/34*–*VQ20 complexes control the expression levels of *MYBs* during male gametogenesis in plants.

## Materials and Methods

### Materials and Plant Growth Conditions

*Arabidopsis thaliana* ecotype Columbia-0 was used as the wild-type in this study. *w34-1* (SALK_133019), *w2-1* (Salk_020399), *vq20-1* (CS827030), *myb97-1* (Salk_112329C), *myb101-2* (Salk_149918C), and *myb120-3* (SALK_063698C) were obtained from the *Arabidopsis* Biological Resource Center (ABRC) and confirmed by PCR using a T-DNA primer and gene-specific primers (Supplementary Table S2). Seeds were surface sterilized and plated on half-strength Murashige and Skoog medium containing 1% sucrose and 0.6% agar. After stratification at 4°C for 3 days, the plates were incubated in an artificial growth chamber at 22°C under a 14-h light/10-h dark photoperiod for 1 week. *Arabidopsis* seedlings were then transferred to nutrient soil and grown in an artificial growth chamber at 22°C under 14-h light/10-h dark or 16-h light/8-h dark photoperiods.

### Transgenic Plants

For the *MYB97-*overexpressing transgenic plants, the coding region of *MYB97* was amplified from *Arabidopsis* ecotype Columbia-0 using primers that contained BamHI and SalI restriction enzyme sites. PCR fragments were cloned into the vector pOCA28 under the control of a pollen-specific expression gene (AT5G09500) promoter (Honys and Twell, 2004; Winter et al., 2007). Transgenic plants were selected using 50 mg/mL kanamycin. The primers used are listed in Supplementary Table S2.

### Isolation of Pollen Grains

Pollen isolation was performed as described previously with some modifications (Honys and Twell, 2003, 2004). To obtain mature pollen, inflorescences collected from over 500 plants were placed in a large plastic pipe. After adding 30 mL of ice-cold 0.3 M mannitol, the flask was shaken vigorously three times for 30 s. The pollen suspension was sequentially filtered through 100- and 53-μm nylon meshes. Pollen grains were concentrated in 1.5-μL Eppendorf tubes by repeated centrifugation steps at 700 × *g* for 5 min at 4°C. The final compact pollen pellet was stored at -80°C. For the three stages of immature male gametophytes, after collection and concentration, the spores were loaded onto the top of 25%/45%/80% Percoll step gradient in a 10-mL centrifuge tube and centrifuged 450 × *g* for 5 min at 4°C. Three subfractions of immature pollen were obtained: microspores; microspores and bicellular pollen mixture; and bicellular pollen. Spores in each fraction were concentrated in Eppendorf tubes by centrifugation at 700 × *g* for 5 min at 4°C and stored at -80°C.

### RNA Extraction and qRT-PCR

Total RNA was extracted from *Arabidopsis* pollen grains using TRIzol reagent (Invitrogen) and used for oligo (dT)_18_-primed cDNA synthesis according to the Fermentas’ reverse transcription protocol (Lei et al., 2017). qRT-PCR was then performed using a SYBR Premix Ex *Taq* kit (Takara) on a Light-Cycler 480 real-time PCR machine (Roche) (Jiang et al., 2014). Three biological replicates of all qRT-PCR amplifications were performed with different pollen grains, and the *ACTIN2* gene was used as a control. Student’s *t*-test was used for the analysis of statistical significance. All primers are listed in Supplementary Table S2.

### Yeast One-Hybrid Assay

The yeast one-hybrid assay was performed as described previously with some modifications (Vidal and Legrain, 1999; Ding et al., 2013). Three fragments (PF1, PF2, and PF3) enriched with two adjacent W-boxes and three corresponding mutant fragments (MPF1, MPF2, and MPF3) with mutant W-boxes of the promotor of *MYB97* were independently cloned into the pAbAi vector (Clontech). pAbAi constructs were then separately integrated into the genome of the Y1HGold strain by homologous recombination to generate six bait reporter strains. *WRKY2-* and *WRKY34-* coding regions were fused to the pGADT7 prey vector (Clontech). For the yeast transformation, pGADT7-WRKY2, pGADT7-WRKY34, and pGADT7 were independently transformed into six bait reporter strains using yeast transformation system (Clontech). Transformations were plated onto SD media (-Leu) to select for transformed cells and incubated at 28°C for 4 days. Large healthy colonies were selected from the transformed and control cells. Each colony was resuspended in 0.9% NaCl, and the OD_600_ adjusted to ∼0.002. Then, the suspensions were diluted in a 10×series and 100 μL of the dilution series was spread on plates containing SD (-Leu) and SD (-Leu) supplemented with 150 ng/mL Aureobasidin A (AbA) Clontech. The plates were then incubated for 3 days at 28°C.

### Transient Expression Assay

In preparation for the assay, the cDNA sequences of WRKY2 and WRKY34 were amplified and independently cloned into pGreenII 62-SK under the control of the 35S promoter to create the effectors (Hellens et al., 2005). At the same time, the cDNA sequence of VQ20 was amplified and cloned into pGreenII62-SK. A 3,062 bp *MYB97* promoter and a 3,062 bp mutant *MYB97* promoter (MPF1 and MPF3) were amplified and fused respectively into pGreenII 0800-LUC vector to generate reporters (Hellens et al., 2005). The preparation of *Arabidopsis* mesophyll protoplasts from wild-type (Col-0) leaves and subsequent transfections were performed as described by Yoo et al. (2007). The effectors and corresponding reporters were cotransformed into prepared *Arabidopsis* mesophyll protoplasts to measure firefly luciferase (LUC) and renilla luciferase (REN) activities using a dual-luciferase reporter assay system (Promega). The relative REN activity was used as an internal control, and LUC/REN ratios were calculated. All of the primers are listed in Supplementary Table S2.

### Cytological and Phenotypic Analyses

Siliques and flowers were examined and photographed with a Leica stereoscopic microscope. Pollen viability was examined using fluorescein diacetate (FDA) staining as described in a previous study (Heslop-Harrison and Heslop-Harrison, 1970). Images of Alexander-stained tissues were obtained under bright light, and GFP-tagged tissues were obtained using a fluorescence microscope (Leica, DM2500) (Lei et al., 2017).

### Germination Assays of Pollen Grains

Pollen germination was assayed *in vitro* by placing pollen grains on plates containing germination buffer solidified with low-melting-point agarose as described previously (Fan et al., 2005). The pollen grains spread on the agar plates were cultured immediately at 23°C and 100% relative humidity. After 6 h, the pollen grains were observed and photographed with a Leica microscope. At least 200 pollen grains and 60 pollen tubes were examined per culture and used to calculate the average germination rate and average pollen tube length, respectively, with Image J software.

To assay *in vivo* pollen germination, emasculated pistils were pollinated as described previously (Ishiguro et al., 2001). The pollinated pistils were collected 8 h after pollination and fixed in ethanol:acetic acid (3:1) for 2 h at room temperature. The fixed pistils were washed three times with distilled water and then maintained in a softening solution of 8 M NaOH overnight. The pistil tissues were subsequently washed in distilled water and stained in aniline blue solution (0.1% aniline blue in 0.1 M K_2_HPO_4_-KOH buffer, pH 11) for 4 h in the dark. The stained pistils were observed and photographed with a fluorescence microscope (Leica).

## Results

### Expression Patterns of *WRKY34*, *WRKY2*, and *VQ20* Overlap With the Three *MYBs* During Male Gametogenesis

**FIGURE 1:**
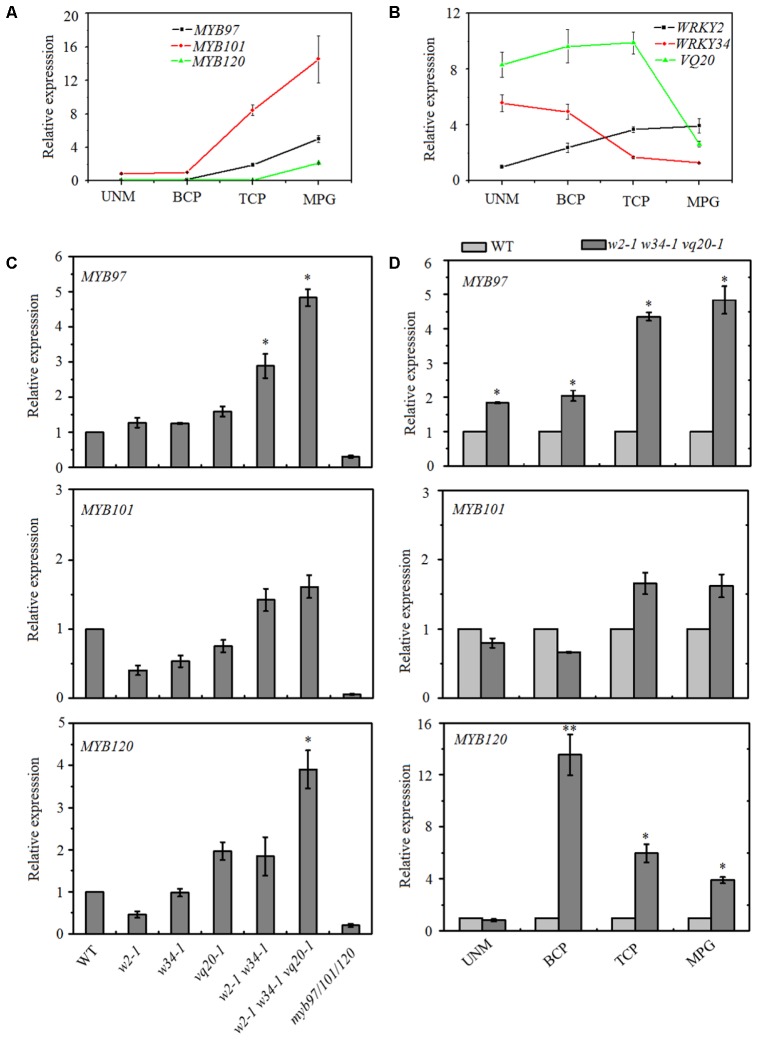
Expression patterns of *MYB97*, *MYB101*, and *MYB120* during male gametogenesis. **(A)** Relative RNA expression levels of *MYB97*, *MYB101*, and *MYB120* during male gametogenesis. **(B)** Relative RNA expression levels of *WRKY2*, *WRKY34*, and *VQ20* during male gametogenesis. **(C)** Relative RNA expression levels of *MYB97*, *MYB101*, and *MYB120* in *w2-1*, *w34-1*, *vq20-1*, *w2-1 w34-1*, *w2-1 w34-1 vq20-1*, and *myb97/101/120* in mature pollen compared to that in wild-type. **(D)** Relative RNA expression levels of *MYB97*, *MYB101*, and *MYB120* during UNM (uninucleate microspores stage), BCP (bicellular pollen stage), TCP (tricellular pollen stage), and (mature pollen) MPG of pollen development (*n* = 3, means ± SD, ^∗^*P* < 0.05, ^∗∗^*P* < 0.01).

Our previous results found that theVQ20 interacts with WRKY2 and WKRY34, and the WRKY2–VQ20 and WRKY34–VQ20 interactions affect the transcriptional functions of WRKYs and are required for pollen development (Lei et al., 2017). However, downstream target genes and mechanisms underlying the *WRKY2/34*–*VQ20* mediated during male gametogenesis in plants are unknown. Therefore, we compared our previous *wrky2-1 wrky34-1 vq20-1* (*w2-1 w34-1 vq20-1*) mature pollen microarray data with previously published pollen microarray data (Liang et al., 2013; Lei et al., 2017). We found that six significantly altered genes in the *myb97-1 myb101-2 myb120-3* triple mutant (designated as *myb97/101/120*) showed opposite changes in the *w2-1 w34-1 vq20-1* triple mutant (Supplementary Table S1). We then examined the expression patterns of *WRKY2*, *WRKY34*, *VQ20*, *MYB97*, *MYB101*, and *MYB120* during male gametogenesis using publicly availabe microarray data, and the data indicated that the *WRKY34* and *VQ20* expression patterns had opposite trends as those of the three *MYBs* during this process (Supplementary Figure S1). We further confirmed this result using quantitative RT-PCR (qRT-PCR), transcripts of *WRKY34* and *VQ20* decreased gradually during male gametogenesis and declined markedly in mature pollen, while the expresssion levels of the three *MYBs* increased gradually through the developmental process and peaked in mature pollen (**Figures 1A,B**). These contrasting expression patterns prompted us to hypothesize that *MYB97*, *MYB101*, and *MYB120* are the potential target genes of *WRKY34* and *WRKY2* during male gametogenesis, and the overlapping expression of *WRKY34*, *WKRY2*, *VQ20* and the *MYBs* suggests that *WRKY34/2* (*VQ20*) may regulate *MYB* transcription during male gametogenesis.

### *WRKY34*, *WRKY2*, and *VQ20* Regulate the Expression Levels of *MYBs* During Male Gametogenesis

**FIGURE 2:**
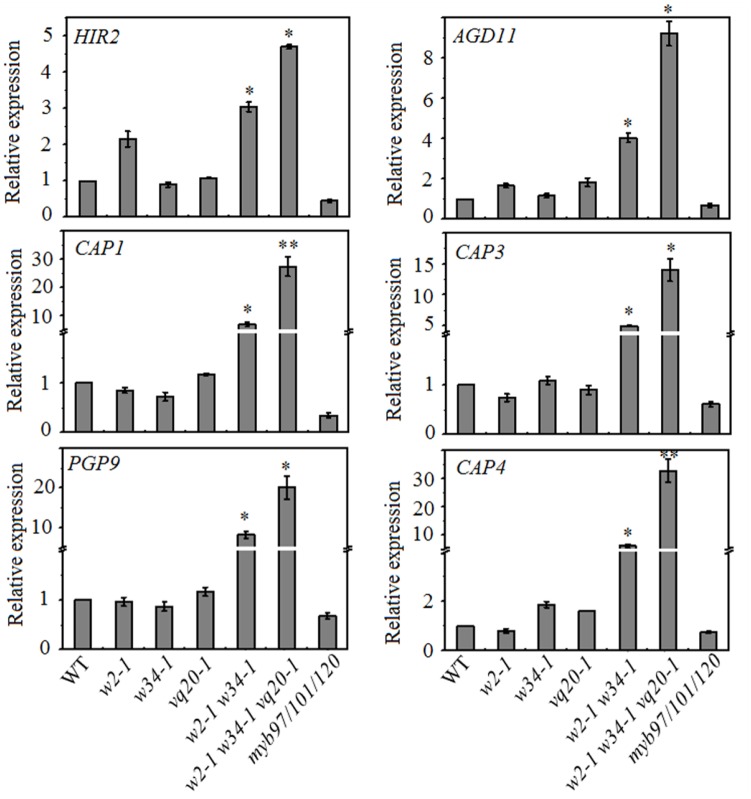
Mutations of *WRKY2*, *WRKY34*, and *VQ20* affect the downstream genes of *MYB97/101/120.* qRT-PCR analysis of six downstream genes of *MYB97/101/120*. These genes are up-regulated in *w2-1 w34-1* mutant and *w2-1 w34-1 vq20-1* triple mutant. Total RNA was isolated from mature pollen; data were normalized to *ACTIN2*. Three independent experiments were performed with similar results. Error bars indicate SD (*n* = 3, ^∗^*P* < 0.05, ^∗∗^*P* < 0.01).

To test this hypothesis, we collected mature pollen grains from wild-type, *wrky2-1* (*w2-1*), *w34-1*, *vq20-1*, *w2-1 w34-1* double mutant, *w2-1 w34-1 vq20-1* triple mutant and *myb91/101/120* triple mutant plants and examined the expression levels of *MYB97*, *MYB101*, and *MYB120* using qRT-PCR. qRT-PCR results showed that the single, double, and the triple mutants conferred very different effects on the expression levels of *MYB97*, *MYB101*, and *MYB120* in mature pollens. The expression levels of *MYB97* and *MYB120* were up-regulated in the *w2-1 w34-1* double mutant and were more significantly increased in the *w2-1 w34-1 vq20-1* triple mutant plants (**Figure 1C**). There was only a slight rise in *MYB101* expression in the *w2-1 w34-1* double mutant plants and the *w2-1 w34-1 vq20-1* triple mutant plants, and no remarkable changes in its expression were observed in the single mutants (**Figure 1C**). Thus, *WRKY34*, *WKRY2*, and *VQ20* suppress the expression of *MYB97*, *MYB101*, and *MYB120*. To further detect the influence of *WRKY34/2* and *VQ20* on *MYBs*, we examined the temporal expression patterns of the *MYBs*, revealing that *MYB97*’s expression level is higher in the *w2-1 w34-1 vq20-1* triple mutant plants than in wild-type throughout male gametogenesis (**Figure 1D**). The expression of *MYB120* was significantly higher in the triple mutant than wild-type during the bicellular pollen stage (BCP), tricellular pollen stage (TCP), and in mature pollen grains (MPG), while *MYB101* expression was slightly increased in TCP and MPG (**Figure 1D**). To confirm the regulatory effects of WRKY34/2–VQ20 on the three *MYBs* during male gametogenesis, we also detected the expression level of eight putative downstream target genes of *MYB97/101/120* in the WRKY/VQ single mutants, *w2-1 w34-1* double mutant, *w2-1 w34-1 vq20-1* triple mutant and *myb97/101/120* triple mutant plants. Six out of the eight selected genes were expressed highest in *w2-1 w34-1 vq20-1* triple mutant plants, followed by *w2-1 w34-1* double mutant plants when compared with the wild-type, whereas they were significantly reduced in *myb97/101/120* triple mutant plants (**Figure 2**). The results verified the changes in the transcript levels of *MYB97*, *MYB10*, and *MYB120* in the *w2-1 w34-1* and *w2-1 w34-1 vq20-1* triple mutant plants. Thus, *WRKY2*, *WRKY34*, and *VQ20* appear to negatively regulate the three *MYBs* during male gametogenesis.

### WRKY2 and WRKY34 Directly Bind to the Promoter of *MYB97*

**FIGURE 3:**
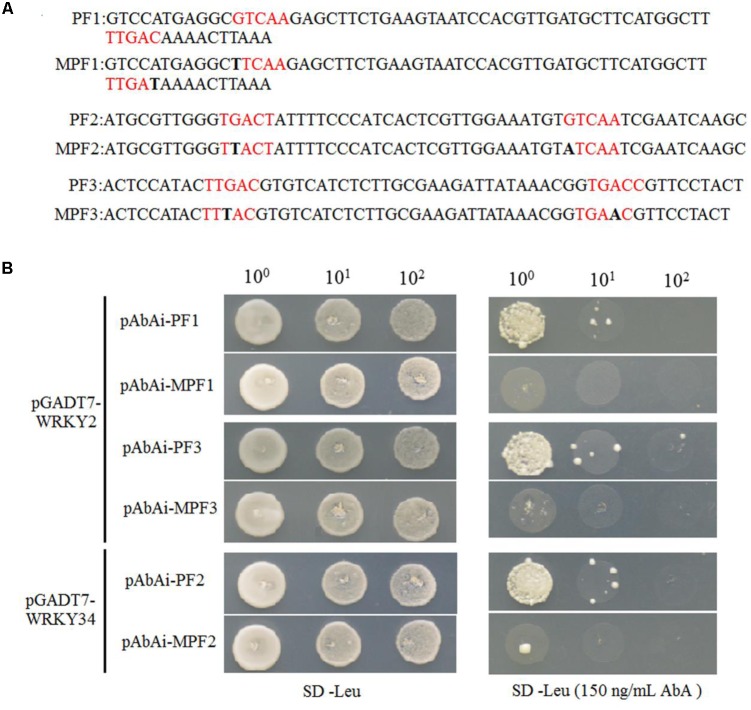
WRKY2 and WRKY34 directly bind to *MYB97* promoter through W-boxes in yeast. **(A)** Potential W-boxes elements were marked in red and Black bold were indicated as the mutant sites. The promoter fragments (short for PF) were indicated. **(B)** Six Bait reporter strains were transformed with a prey vector (containing WRKY2 and WRKY34 fused to a GAL4 activation domain). Cells were spotted on media on selective media lacking Leu and selective media lacking Leu supplemented with 150 ng/mL AbA to suppress background growth.

WRKY proteins regulate the expression levels of downstream target genes by binding directly to W-boxes in their promoters (Eulgem et al., 2000; Zhang and Wang, 2005; Rushton et al., 2010; Chen et al., 2012). Because *WRKY2* and *WRKY34* repress the expression of *MYB97*, *MYB101*, and *MYB120* during male gametogenesis, we investigated whether these WRKY transcription factors might directly bind to W-boxes in the *MYB* promoters. There are 13 W-boxes with TGAC core sequences in the 2.7 kb *MYB97* promoter, which harbors three W-boxes groups containing two adjacent W-box. We conducted a yeast one-hybrid assays to test whether the W-box-enriched region of the promotor (containing two adjacent W-boxes) was the binding site for WRKY34 and/or WRKY2. First, we cloned this enriched region into a pBait-AbAi plasmid and generated a bait reporter strain by introducing the construct into the genome of theY1H Gold strain using homologous recombination. We also constructed negative control bait reporter strains that contained, three groups of mutant W-boxes, using the same method (**Figure 3A**). The associations between *WRKY2* (*WRKY34*) and these three promoter fragments were confirmed by cotransformation on selective media lacking Leu supplemented with, concentrations of up to 150 ng/mL of AbA, which is toxic to yeast at low concentrations was added to the media to suppress background activation (**Figure 3B**). The bait reporter strains with the natural promoter regions (PF1 and PF3) grew well in the selective media when transformed with *WRKY2*, but the strains containing the corresponding mutant promoter fragments (MPF1 and MPF3) did not. WRKY34 was able to bind to the PF2 of the *MYB97* promoter but not to the corresponding mutant promoter (**Figure 3B**). The results indicated that WRKY2 and WRKY34 were able to bind these regions in the *MYB97* promoter.

**FIGURE 4:**
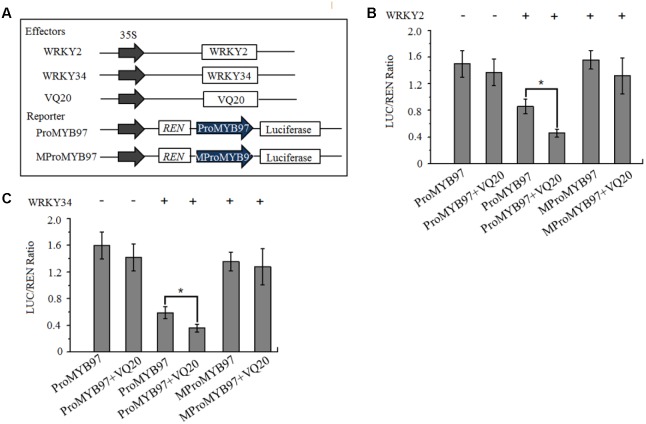
WRKY2 and WRKY34 directly bind to *MYB97* promoter *in vitro*. **(A)** The schematic diagram of the constructs used in the transient expression assays of **(B,C)**. **(B,C)** Transient expression assay shows that suppression of MYB97 by WRKY2 and WRKY34, and promote this suppression by VQ20. The ProMYB97-LUC reporter (or MProMYB97-LUC reporter) was cotransformed with the indicated constructs. Three independent experiments were performed with similar results. Error bars indicate SD (*n* = 3, ^∗^*P* < 0.05).

We then used a dual luciferase reporter in *Arabidopsis* mesophyll protoplasts to confirm whether the binding of WRKY2 and WRKY34 to the *MYB97* promoter depended on W-boxes. In this assay, *WRKY2* and *WKRY34* under the cauliflower mosaic virus (CaMV) 35S promoter were used as effectors. The reporter consisted of 35S promoter-driven *REN*, as an internal control, and LUC driven by either a normal *MYB97* promoter (ProMYB97)or a *MYB97* promoter with mutated W-boxes (MProMYB97; **Figure 4A**) (Hellens et al., 2005; Lei et al., 2017). As shown in **Figures 4B,C**, the transformation of *WRKY2* and *WRKY34* inhibited the reporter activity driven by the *MYB97* promoter, but not the mutant *MYB97* promoter, which had mutations in W-boxes s1-s13. The coexpression of *VQ20* and *WRKY2* (or *WRKY34*) dramatically attenuated ProMYB97-LUC activity but did not change the ratio of MproMYB97-LUC/REN activity. Thus the region of the *MYB97* promoter containing the W-boxes appears to be critical for its interaction with WRKY34 or WKRY2, and that VQ20, as a partner of WRKY2 and WRKY34, assists in *WKRY* functioning.

### *MYB97* Overexpression Results in Male Fertility Defects

**FIGURE 5:**
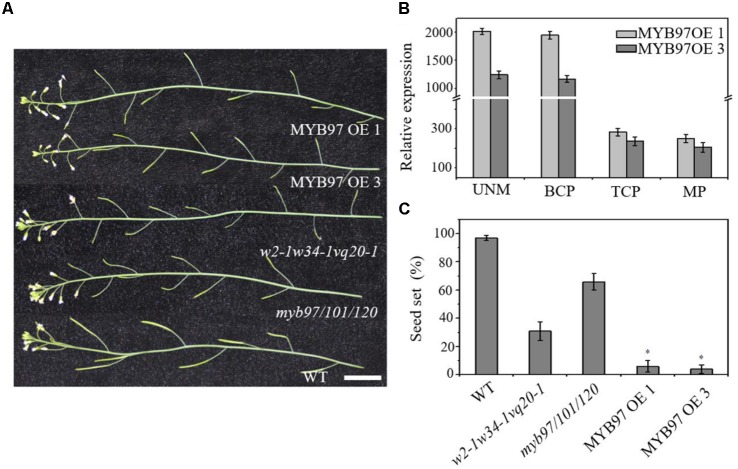
Characterization of the *MYB97*-overexpression transgenic plants. **(A)** Inflorescences from WT, *myb97/101/120*, *w2-1 w34-1 vq20-1*, MYB97OE 1, and MYB97OE 3 plants. Bar = 2 cm. **(B)** Relative RNA expression levels of MYB97OE1 and MYB97OE 3 during UNM (uninucleate microspores stage), BCP (bicellular pollen stage), TCP (tricellular pollen stage), and (mature pollen) MPG of pollen development (*n* = 3, means ± SD). **(C)** The seed set rate of WT, *myb97/101/120*, *w2-1 w34-1 vq20-1*, MYB97OE 1, and MYB97OE 3 plants. 30 siliques were examined for each sample. All measurements represent the average of three biological replicates. Error bars indicate SD (*n* = 3, ^∗^*P* < 0.05).

The expression of *MYB97* was up-regulated during each phase of male gametogenesis in *w2-1 w34-1 vq20-1* compared with the wild-type. To assess the influence of this elevated *MYB97* expression on pollen development, we generated 17 *MYB97* overexpression lines (namely, MYB97OE) and chose 2 lines (lines #1 and #3) as the representatives. These two MYB97OE lines exhibited reduced fertility and shorter siliques, but most of the vegetative parts appeared normal compared with those of wild-type (**Figures 5A–C**). To investigate whether *MYB97* overexpression affects male or female functions, *MYB97*-overexpression plants were used as male or female parents in crosses with the wild-type plants (**Table 1**). When homozygous *MYB97*-overexpression plants were used as the female parent in a cross with a wild-type plant, the number of seeds per silique was similar to that of the wild-type. In a reciprocal cross, however, only a few ovules in the silique were fertilized and developed into mature seeds (**Table 1**). To determine the sex-related transmission efficiency of heterozygous *MYB97-* overexpression lines, a genetic analysis was performed. When the wild-type plants were used as pollen donors in crosses with heterozygous *MYB97*-overexpression lines, approximately 50% of the resulting progeny having kanamycin resistance (**Table 2**). In contrast, the number of resulting progeny harboring contained kanamycin resistance was significantly reduced when the wild-type plants were used as recipients in crosses with the heterozygous *MYB97*-overexpression lines pollen grains (**Table 2**).

**Table 1:** *MYB97-*overexpression lines exhibit male fertility defects.

Parent (female × male)	Seed set (%)
WT × WT	96.57 ± 1.27
*MYB97OE 1* (self)	**5.22 ± 4.78**
*MYB97OE 1* × WT	97.51 ± 3.42
WT × *MYB97OE 1*	**4.25 ± 3.93**
*MYB97OE 3* (self)	**3.87 ± 2.86**
*MYB97OE* × WT	95.46 ± 3.19
WT × *MYB97OE*	**3.32 ± 2.67**


*The statistical analysis of the seed sets was performed in plants 50 days after transplantation into the soil; 30 siliques were examined for each hybrid combination. Bold numbers indicate significant aberrant seed set from the expected.*

**Table 2:** Genetic transmission ratios of *MYB97-overexpression* heterozygous lines.

Parent (female × male)	Kan^+^	Kan^-^	K^+^:K^-^	TE_F_ (%)	TE_M_ (%)
*MYB97OE 1/-* × WT	132	116	1.138:1	113	–
WT × *MYB97OE 1/-*	7	255	0.027:1	–	**2.7**
*MYB97OE 3/-* × WT	171	179	0.955:1	95.5	–
WT × *MYB97OE 3/-*	2	187	0.069:1	–	**1.0**


*Female transmission efficiency (TE_*F*_), male transmission efficiency (TE_*M*_); not applicable (–). Bold numbers indicate significant aberrant transmission efficiency from the expected ratio of 100%.*

**FIGURE 6:**
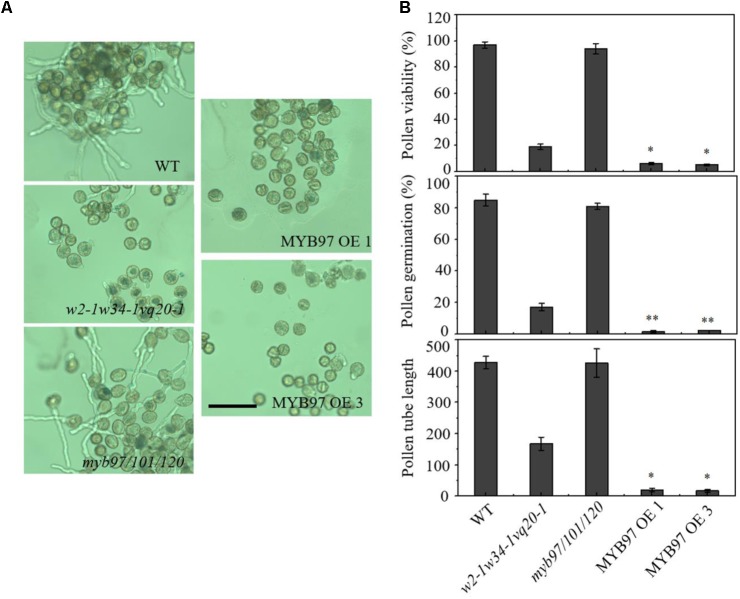
*MYB97* overexpression caused defects of pollen germination, pollen viability, and pollen tube growth. **(A)** Images of pollen grains from WT, *w2-1 w34-1 vq20-1*, *myb97/101/120*, MYB97OE 1, and *w2-1* MYB97OE 3 germinated *in vitro*. Bar = 100 μm. **(B)** Pollen viability ratio by FDA staining, *in vitro* pollen germination ratio, and average pollen tube length in WT, *w2-1 w34-1 vq20-1*, *myb97/101/120*, MYB97OE 1, and *w2-1* MYB97OE 3 plants. All measurements represent the average of three biological replicates. Error bars indicate SD (*n* = 3, ^∗^*P* < 0.05, ^∗∗^*P* < 0.01).

To accurately assess pollen development, we performed FDA staining to determine the pollen viability of *MYB97*-overexpression lines. Many fewer pollen grains of the *MYB97*-overexpression lines showed FDA fluorescence compared with those of the wild-type (**Figure 6B**). We further investigated the pollen germination and tube growth of these *MYB97*-overexpression lines. We hypothesized that the reduced pollen viability may lead to a reduction in pollen germination. As expected, the mean germination ratio and pollen tube length of pollen grains in plants of *MYB97*-overexpression lines were dramatically lower than those of wild-type plants (**Figures 6A,B**). Like the pollen phonotype of the *w2-1 w34-1 vq20-1* triple mutant plants, *MYB97* overexpression lines plants showed male sterility, with defects in pollen germination and pollen tube length. Thus *MYB97* overexpression affected male gametophytic functions.

## Discussion

Gene expression is precisely regulated at the spatiotemporal level during male gametogenesis (Honys and Twell, 2003; Hafidh et al., 2016). During pollen development in *Arabidopsis*, 607 transcription factor genes are expressed (Honys and Twell, 2004). These genes are regulated precisely by a gene regulatory network. In *Arabidopsis*, the germline-specific MYB transcription factor DUO POLLEN1 (DUO1) functions in the development of the male germline and in the transcriptional control of a gene regulatory network (Durbarry et al., 2005; Rotman et al., 2005; Borg et al., 2011). DUO1 expression is controlled by transcriptional regulation during male gametophyte development (Peters et al., 2017). *MYB97*, *MYB101*, and *MYB120* genes function in the later development of male gametophytes (Liang et al., 2013). We found that these *MYBs* are expressed in tricellular pollen and their expression peaks in mature pollen, while transcripts of *WRKY34* and *VQ20* decreased gradually during male gametogenesis and were lowest in mature pollen. The expression levels of *MYB97* and *MYB120* were highest in the *w2-1 w34-1 vq20-1* triple mutant plants, followed by the *w2-1 w34-1* double mutants. Only a slight rise in *MYB101* expression in the *w2-1 w34-1* double mutant plants and the *w2-1 w34-1 vq20-1* triple mutant plants was observed (**Figure 1C**). qRT-PCR results demonstrated that six potential downstream target genes of MYB97/101/120 were markedly up-regulated in the *w2-1 w34-1 vq20-1* triple mutant. Thus, WRKY2/34–VQ20 may suppress the expression levels of *MYB97* and *MYB120* and affect their potential downstream target genes during male gametogenesis. WRKY proteins bind directly to W-boxes in the promoters of their target genes (Yang et al., 1999; Yu et al., 2001; Chen et al., 2013; Li et al., 2016). The transient transformation of *Arabidopsis* mesophyll protoplasts suggested that *WRKY2* and *WRKY34* directly bind to the promoter of *MYB97* (**Figure 4**). *WRKY2* and *WRKY34* directly bind to different fragments of the *MYB97* promoter in yeast cells, which suggested that the binding is specific for WRKY2 and WRKY34. Li et al. (2016) showed that the binding of WRKY12 and WRKY13 to disparate regions of the *FUL* promoter regulates flowering under short-day conditions in *Arabidopsis* (Li et al., 2016). There are five and seven W-boxes in the 1.8-kb promoters of *MYB101* and *MYB120*, respectively, suggesting that *MYB101* and *MYB120* may be regulated by WRKY34 and WRKY2, respectively, during male gametogenesis. We provide evidence that VQ20 assists the function of these WRKY transcription factors and collectively regulates *MYB97* and *MYB120* expression during male gametogenesis.

The uncontrolled regulation of transcriptional factors affects pollen development in *Arabidopsis* (Rotman et al., 2005; Brownfield et al., 2009; Berger and Twell, 2011). For example, the loss of MIKC^∗^ MADS transcription factors affects pollen maturation and tube growth, causing defects in male fertility (Verelst et al., 2007a,b; Adamczyk and Fernandez, 2009). *WRKY34* expression is up-regulated and *MYB97* transcription is down-regulated in *agl65-1 agl66-1 agl104-1* triple mutant plants (Verelst et al., 2007a). Pollen tubes of the *myb97/101/120* triple mutant exhibited uncontrolled growth and failed to discharge the pollen sperm cells (Leydon et al., 2013; Liang et al., 2013). Mutations of *WRKY2*, *WRKY34* and *VQ20* displayed defects in pollen development by influencing the expression levels of multiple genes involved in pollen development, pollen germination and tube growth (Lei et al., 2017). Here, we found that the expression levels of these *MYB*s were not properly regulated during male gametogenesis in the *w2-1 w34-1 vq20-1* triple mutant plants. When MYB97 expression was up-regulated in a wild-type background, these MYB97-overexpression lines had a similar phenotype as the *w2-1 w34-1 vq20-1* triple mutant, with defects in pollen viability, pollen germination, and pollen tube length. These results suggested that the uncontrolled regulation of transcriptional factors may affect pollen development in *Arabidopsis*.

## Accession Numbers

Sequence data from this article can be found in the Arabidopsis Genome Initiative or GenBank/EMBL databases under the following accession numbers: WRKY2 (AT5G56270), WRKY34 (AT4G26440), VQ20 (AT3G18360), MYB97 (AT4G26930), MYB101 (AT2G32460), MYB120 (AT5G55020), DUO1 (AT3G60460), AT5G09500, AT3G19690, AT1G69840, AT5G66300, AT1G69840, AT3G19690, AT3G12580, CAP1 (AT4G34490), CAP2 (AT1G01310), CAP2 (AT3G09590), CAP4 (AT5G02730), AGD11 (AT3G07490), PGP9 (AT4G18050), HIR2 (AT1G69840), AGL30 (AT2G03060), AGL65 (AT1G18750), AGL66 (AT1G77980), AGL104 (AT1G22130), and ACTIN2 (AT3G18780).

## Author Contributions

DY and RL designed the study. RL and ZM performed the research. RL analyzed the data and wrote the article. DY, RL, and ZM revised the article. All authors read and approved the final manuscript.

## Conflict of Interest Statement

The authors declare that the research was conducted in the absence of any commercial or financial relationships that could be construed as a potential conflict of interest. The reviewers LB and SK and handling Editor declared their shared affiliation.
