# Supplementary material for: WRKY2/34–VQ20 Modules in Arabidopsis thaliana Negatively Regulate Expression of a Trio of Related MYB Transcription Factors During Pollen Development
Source: Front Plant Sci. 2018 Mar 19;9:331. doi: 10.3389/fpls.2018.00331 (PMC5867338; doi:10.3389/fpls.2018.00331)
Supplement: Supplementary file 1 [file Table_1.DOCX]

**Table S1.** Compare of chosen genes expression between two microarrays.

| Gene | Gene Title | FC(myb97/101/120 VS WT) | FC(w2 w34 vq20 VS WT) |
| --- | --- | --- | --- |
| AT1G69840 | Hypersensitive-induced response protein 2 | -14.61 | 1.72 |
| AT5G66300 | NAC105(NAC-domain transcription factor) | -5.69 | -3.47 |
| AT3G19690 | Secretory protein | -4.34 | 3.19 |
| AT1G69840 | PHB domain-containing membrane-associated protein | -3.42 | 1.722 |
| AT3G19690 | Cysteine-rich seeretory proteins, Antigen 5, and Pathogenesis-related 1 protein | -2.19 | 3.19 |
| AT3G12580 | HSP70 (heat shock protein) | -1.18 | 1.17 |
| AT3G07490 | AGD11 (ARF-GAP DOMAIN 11) | -1.24 | 2.73 |

FC: Fold change=Log_2_(triple mutant/wild-type)
