# Supplementary material for: WRKY2/34–VQ20 Modules in Arabidopsis thaliana Negatively Regulate Expression of a Trio of Related MYB Transcription Factors During Pollen Development
Source: Front Plant Sci. 2018 Mar 19;9:331. doi: 10.3389/fpls.2018.00331 (PMC5867338; doi:10.3389/fpls.2018.00331)
Supplement: Supplementary file 2 [file Table_2.docx]

**Table S2**. Sequences of the primers used in this study.

| Function | Primers | Sequences (5’-3’) | Enzyme |
| --- | --- | --- | --- |
| Genotyping | LBa1 | TGGTTCACGTAGTGGGCCATCG |  |
|  | WRKY2-LP | TTTTCTTTTTCACACGTTAAGCC |  |
|  | WRKY2-RP | TGTTAGAACACGAATCACCCC |  |
|  | WRKY34-LP | AGCTTGAGCCCAAGTTAAAGC |  |
|  | WRKY34-RP | GCATGTCTTGGCCAGTACCGGATG |  |
|  | KO | AAACGTCCGCAATGTGTTAT |  |
|  | VQ20-1L | TAAGTGAAGATAGTGAATCGTCGTC |  |
|  | VQ20-1R | ATGATCACAGAAAAATTGTTTCGAG |  |
|  | VQ20-2L | GACGGTGGTTTTTTAATAACGTGAG |  |
|  | VQ20-2R | CTAGTGGCGATAATCTCTTAACTGC |  |
|  | MYB97-1L | tcttcctttggtgttaaacctgtg |  |
|  | MYB97-1R | tgtgtcccaaacgttgttataaca |  |
|  | MYB101-2L | ctaacagatgctaggcatgttgct |  |
|  | MYB101-2R | tctccgagaattcggacaca |  |
|  | MYB120-3L | AACTCCATCCACATCCACATC |  |
|  | MYB120-3R | TGTTGTGGTTCGGAAGAGAAG |  |
| Transgenic | ProS19-97-F | TATGAGCTCGCCGAACAAGGCATCTTCTTC | SacI |
|  | Pro S19-97-R | TATGGATCCtgtttgtttggaattgctgaa | BamHI |
|  | ProMYB97-97-3 | TATGGATCCATGATCGTGTACGGTGGGG | BamHI |
|  | ProMYB97-97-4 | TATGTCGACCTAGCAGATCCCTGGCAAGT | SalI |
| Dual-Luc | pG-62-SK-VQ20-1 | TATGGATCCATGAGCTCAACGTACAAG | BamHI |
|  | pG-62-SK-VQ20-2 | TATGAATTCTTAAAAATCGCGAAACTC | EcoRI |
|  | P0800-PMYB97-1 | TATGTCGACTCGCAAATATTTGATACAGGTTA | SalI |
|  | P0800-PMYB97-2 | TATGGATCCTGCCCCCTCTGCGTCCCG | BamHI |
|  | P62-SK-WRKY34-F | TATGAATTCATGGCTGGTATTGATAATAAAG | EcoRI |
|  | P62-SK-WRKY34-R | TATGGTACCTCATATCTGTCGTAATCTACTCA | KpnI |
|  | P62-SK-WRKY2-F | TATGAATTCATGGCTGGTTTTGATGAAAAT | EcoRI |
|  | P62-SK-WRKY2-R | TATGGTACCTCAAATCTGAGGTAATCTAC | KpnI |
| qRT-PCR | qVQ20F | GGTGCTTATTTACCGACATTTCC |  |
|  | qVQ20R | AAAGAGCTTCTCATATTAGGCGC |  |
|  | qW34F | CCCTGACTTACGATGGAGAGGT |  |
|  | qW34R | ACAACTCTTGGCTCACGGCT |  |
|  | qMYB97F | GGAGGTTTCGGACATCGTGT |  |
|  | qMYB97R | TCTTGCATTGTGTTCATCTGGA |  |
|  | qMYB101F | GGCGGACTCTTCAAGGAC |  |
|  | qMYB101R | GTTGTGAATATTAGGGTTTGCTC |  |
|  | qMYB120F | GTAACAAATGGGCTCGCATG |  |
|  | qMYB120R | GGATGGAGTTGATGGTTAGGG |  |
|  | qHIR2-F | GAGCCTGATGTGCACGTCAA |  |
|  | qHIR2-R | TGCCTCAGCTTTCTCACTCG |  |
|  | qCAP1-F | TGAGTAGTGGAGAAATGTCGGC |  |
|  | qCAP1-R | TAATGTAGACAGGTGCCACCGT |  |
|  | qPGP9-F | TTAGTTTCCGATACCCAATGCG |  |
|  | qPGP9-R | ATGCTTATCACCGTTGATTTCC |  |
|  | qAGD11-F | ACGTTGATGGAGATGGCATG |  |
|  | qAGD11-R | CCTAAGGCGGCAAATCCA |  |
|  | qCAP3-F | GCGTTTGGTAAACAGAGCGA |  |
|  | qCAP3-R | TTGGCACGCCTGAAGAAA |  |
|  | qCAP4-F | CGCCGAGGAGAGTTGTTGAT |  |
|  | qCAP4-R | TGGTTGTACGCCAAACGATCT |  |
|  | qLSU4-F | ACGGAGAGATGGAGAAAGCG |  |
|  | qLSU4-R | CGGCAAGCTGAGAGCAAAGA |  |
|  | qXTH4-F | TGGTTGCCAAGCTTCTGTGG |  |
|  | qXTH4-R | AGCGTCAAGGTCACGGAACT |  |
|  | ACTIN-Q-1 | TGTGCCAATCTACGAGGGTTT |  |
|  | ACTIN-Q-2 | TTTCCCGCTCTGCTGTTGT |  |
