# Supplementary material for: WRKY2/34–VQ20 Modules in Arabidopsis thaliana Negatively Regulate Expression of a Trio of Related MYB Transcription Factors During Pollen Development
Source: Front Plant Sci. 2018 Mar 19;9:331. doi: 10.3389/fpls.2018.00331 (PMC5867338; doi:10.3389/fpls.2018.00331)
Supplement: Supplementary file 3 [file Data_Sheet_1.docx]

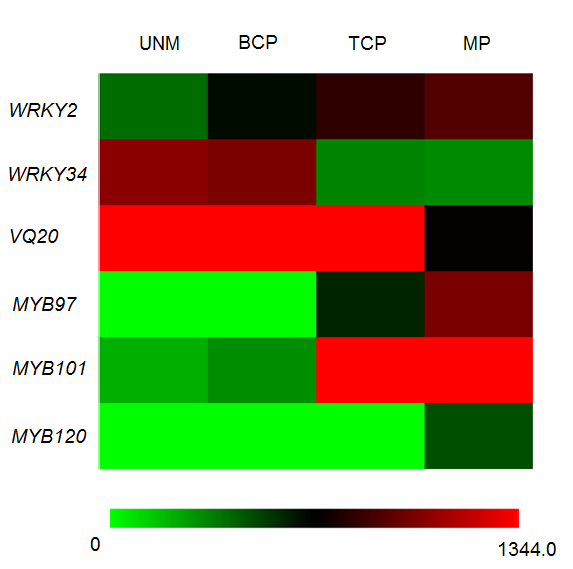


**Supplemental Figure 1.** Expression pattern of *WRKY2*, *WRKY34*, *VQ20*, *MYB97*, *MYB101* and *MYB120* during male gametogenesis. The public microarray data (Honys and Twell, 2004) compiled using the BAR HeatMapper tool.
